# Protection Evaluation of a Five-Gene-Deleted African Swine Fever Virus Vaccine Candidate Against Homologous Challenge

**DOI:** 10.3389/fmicb.2022.902932

**Published:** 2022-07-29

**Authors:** Zhenhua Xie, Yingnan Liu, Dongdong Di, Jingyi Liu, Lang Gong, Zongyan Chen, Yao Li, Wanqi Yu, Lu Lv, Qiuping Zhong, Yingying Song, Xinxin Liao, Qingqing Song, Heng Wang, Hongjun Chen

**Affiliations:** ^1^Shanghai Veterinary Research Institute, Chinese Academy of Agricultural Sciences (CAAS), Shanghai, China; ^2^Biosafety Research Center, Chinese Academy of Agricultural Sciences (CAAS), Shanghai, China; ^3^The Spirit Jinyu Biological Pharmaceutical Co., Ltd., Hohhot, China; ^4^Guangdong Provincial Key Laboratory of Prevention and Control for Severe Clinical Animal Diseases, College of Veterinary Medicine, South China Agricultural University, Guangzhou, China

**Keywords:** African swine fever virus, recombinant viruses, live attenuated vaccine, safety, protective efficacy

## Abstract

African swine fever virus (ASFV) represents a serious threat to the global swine industry, and there are no safe or commercially available vaccines. Previous studies have demonstrated that inactivated vaccines do not provide sufficient protection against ASFV and that attenuated vaccines are effective, but raise safety concerns. Here, we first constructed a deletion mutant in which *EP153R* and *EP402R* gene clusters were knocked out. Based on the deletion mutant, a further deletion from the *MGF_360-12L, MGF_360-13L* to *MGF_360-14L* genes was obtained. The five-genes knockout virus was designated as ASFV-ΔECM3. To investigate the efficacy and safety of the ASFV-ΔECM3 virus as a vaccine candidate, the evaluation of the virus was subsequently carried out in pigs. The results showed that the ASFV-ΔECM3 virus could induce homologous protection against the parental isolate, and no significant clinical signs or viremia were observed. These results show that the contiguous deletion mutant, ASFV-ΔECM3 encompassing the *EP153R*/*EP402R* and *MGF_360-12L/13L/14L* genes, could be a potential live-attenuated vaccine candidate for the prevention of ASFV infection.

## Introduction

African swine fever (ASF), a fatal hemorrhagic and highly contagious disease in swine caused by the African swine fever virus (ASFV), has led to significant economic losses in the global swine industry (Sánchez-Cordón et al., 2018). Different isolates of ASFV exhibit differential clinical manifestations and pathological changes, leading to 100% mortality (Sánchez-Vizcaíno et al., 2015; Rodríguez-Bertos et al., 2020).

African swine fever virus is a large double-stranded DNA virus of the family Asfarviridae. The viral particle presents an icosahedral morphology, with a genome size between 170 and 193 kb, encoding more than 150 proteins (Dixon et al., 2013; Andrés et al., 2020). The ASFV C-type lectin protein is encoded by the *EP153R* gene, which is involved in the hemadsorption process observed in ASFV-infected cells (Galindo et al., 2000). In addition, it has been shown to play major roles in the modulation of MHC class I antigen presentation and inhibition of apoptosis (Hurtado et al., 2004, 2011). The ASFV *EP402R* gene, downstream from *EP153R*, is a transmembrane glycoprotein that is similar to host CD2 (Rodríguez et al., 1993). It is essential for hemadsorption and the inhibition of lymphocyte proliferation (Borca et al., 1994, 1998).

The ASFV pandemic has caused tremendous economic losses to the swine industry. However, there is currently no effective vaccine or antiviral drug for ASFV at present. Effective and safe vaccines are desperately required but still unavailable. For several years, multiple vaccine development strategies, including those based on inactivated, live attenuated, subunit, vectored, and DNA vaccines have been evaluated in swine (Sang et al., 2020). Currently, the most promising strategies for developing effective vaccines are based on live-attenuated vaccines. For example, the deletion of the *MGF_360, MGF_505* genes provide reliable protection against homologous virulent viruses (O'Donnell et al., 2015; Reis et al., 2016). The deletion of the *EP402R* gene from ASFV BA71 isolate provides protection against both homologous virulent viruses and heterologous ASFV challenges (Monteagudo et al., 2017). In addition, the deletion of multiple genes has been performed to improve vaccine safety (O'Donnell et al., 2016a; Chen et al., 2020; Teklue et al., 2020). Studies have found that the deletion of the *EP402R* and *EP153R* genes from ASFV-G-Δ9GL fails to protect them against challenges with parental virulent ASFV Georgia 2007 isolate, but they may increase vaccines safety (Gladue et al., 2020). Recent studies have shown that the deletion of the *EP402R* and *EP153R* genes from BeninΔDP148 plays a synergistic role in reducing clinical signs and levels of virus in the blood. These results show that the deletion of *EP153R* and *EP402R* may further attenuate the virus and increase vaccine safety (Petrovan et al., 2022). Based on this trial, the continued evaluation of contiguous deletion encompassing the *EP153R* and *EP402R* genes in ASFV vaccines is warranted.

Here, we constructed a deletion mutant ASFV by targeting the *EP153R* and *EP402R* genes of the ASFV Chinese strain GZ201801. Based on the deletion mutant, a further deletion from the *MGF_360-12L, MGF_360-13L* to *MGF_360-14L* genes was obtained. The efficacy and safety of this live-attenuated vaccine were subsequently evaluated in pigs.

## Materials and Methods

### Virus and Cells

Primary bone marrow-derived macrophages (BMDMs) were collected from specific-pathogen-free (SPF) pigs as described previously (Liu et al., 2021). BMDMs cells were cultured in RPMI 1640 (Gibco, Thermo Fisher Scientific, Waltham, MA, USA) containing 10 % fetal bovine serum (FBS), 2 mM L-glutamine (Gibco, Thermo Fisher Scientific, Waltham, MA, USA), 1% Pen-strep and 10 ng/ml rpGM-CSF (R&D Systems, Minneapolis, MN, USA). The ASFV strain GZ201801 (GenBank accession number: MT496893.1) belonged to the genotype II and was isolated from a piglet with severe infection in Guangdong (Ji et al., 2021).

### Virus Titration

Bone marrow-derived macrophages cells were infected with the ASFV-ΔECM3 virus or the parental GZ201801 wild-type (WT) virus, respectively. The preformed monolayers were prepared in six-well plates and infected at an MOI of 0.01. The GZ201801 virus was titrated by limiting dilution in cultures of BMDMs cells using a monoclonal antibody (mAb) against p30 protein (prepared in our lab). Briefly, 10-fold serial dilutions of viruses were added to 96-well plates. After 4 days, the cells were fixed with 4% paraformaldehyde and permeated with 0.1% Triton X-100 for 10 min, and washed with PBS. The cells were blocked with 3% BSA for 1 h and then incubated with p30 mAb for 1 h at 37°C and subsequently with Alexa488-labeled anti-mouse secondary antibody IgG (H+L) for 30 min at 37°C. Finally, the cells were observed under a fluorescence microscope (EVOS M5000, Thermo Fisher Scientific, Waltham, MA, USA). The ASFV-ΔECM3 was directly observed under a fluorescence microscope. Virus titers were calculated by the Reed and Muench method (Reed and Muench, 1938).

### Construction of Recombinant Virus

The genes were knocked out and generated by homologous recombination by infection with parental virus GZ201801 and transfection with the recombination transfer vectors (p72eGFPΔEP153R/ΔEP402R and p72mCherryΔMGF_360-12L/13L/14L) described in our previous study (Liu et al., 2021). The recombinant transfer vector p72eGFPΔEP153R/ΔEP402R contains the upstream and downstream fragments (about 1,200 bp each) of the target deletion *EP153R*-*EP402R* gene with the p72 promoter and eGFP gene in the middle (Borca et al., 2017). The recombinant transfer vector p72mCherryΔMGF_360-12L/13L/14L contains the upstream and downstream fragments (about 1,200 bp each) of the target deletion *MGF_360-12L/13L/14L* gene with the p72 promoter and mCherry gene in the middle. The recombinant transfer vector was obtained by DNA synthesis (Shanghai Generay, Shanghai, China). BMDMs were transfected with 2 μg of the recombinant transfer vector p72eGFPΔEP153R/ΔEP402R using jetPEI-macrophage transfection (Polyplus-transfection Inc., Illkirch, France) and infected with the GZ201801 virus at an MOI of 1 at 6 h post-transfection. After 48 h, ASFV-ΔEP153R/ΔEP402R viruses were purified in BMDMs by limiting dilution assay. In brief, the viruses were serially diluted in 96-well plates. After virus dilution, the green fluorescent cells were picked by limited dilution. BMDMs were transfected with the p72mCherryΔMGF_360-12L/13L/14L plasmid and infected with the ASFV-ΔEP153R/ΔEP402R virus at an MOI of 1 at 6 h post-transfection. After 48 h, the viruses were purified in BMDMs by limiting dilution assay. The cells exhibiting both green and red fluorescence were picked by limited dilution.

### PCR Confirmation and Next-Generation Sequencing

The ASFV-ΔECM3 deletion mutant virus was confirmed by PCR. Detection of the *EP153R, EP402R, MGF_360-12L, MGF_360-13L*, and *MGF_360-14L* genes was confirmed by PCR using the specific primers listed in Table 1. For the whole-genome next-generation sequencing, genomic DNA was extracted from BMDMs infected with ASFV-ΔECM3 separately. The extracted DNA was sequenced using Illumina NovaSeq 6000, PE150 (Tanpu Biotechnology Co. Ltd, Shanghai, China).

**Table 1:** Primer sequences.

**Primer name**	**Sequence (5^**′**^-3^**′**^)**	**Gene**
*EP153R-F*	ATGTTTTCTAACAAAAAGTACATCGGTCT	*EP153R*
*EP153R-R*	TTATTTACTACAAATATATAATAAACTT ACATGTTTTTGTTTTTTGT	
*EP402R-F*	CCTAAGCCTTACAGTCGTTATCAG	*EP402R*
*EP402R-R*	TGGCGGGATATTGGGTAGTA	
*MGF_360-12L-F*	TGCCCACGAACCAACATTA	*MGF_360-12L*
*MGF_360-12L-R*	GTGGTGGCCGGACTATAAAT	
*MGF_360-13L-F*	GGATCGTGGCCGAATACAAATA	*MGF_360-13L*
*MGF_360-13L-R*	CAAAGGCATTACCACCCAAATC	
*MGF_360-14L-F*	CGGGTAGCTTGTAGCCTTTATT	*MGF_360-14L*
*MGF_360-14L-R*	GATACTCTTCGGCTCGTTTCAG	

### Virus Growth Analysis

The virus growth curves of the ASFV strain GZ201801 and ASFV-ΔECM3 virus were analyzed. The cells were seeded in six-well plates and infected at an MOI of 0.01. After 1 h of adsorption at 37°C under 5% CO_2_, the inoculum was removed, and the cells were rinsed twice with PBS buffer. The cell pellets and supernatants were collected at 0, 24, 48, 72, and 96 h post-infection (hpi) and freeze-thawed three times. Virus titers were calculated as described previously.

### Animal Experiments

#### Experiment 1

Safety investigation. Piglets weighing about 15–20 kg were purchased from a local farm with high biosecurity and hygiene standards. The ASFV-ΔECM3 virus was assessed for its virulence phenotype relative to the virulent parental ASFV GZ201801 virus. Two groups of pigs (*n* = 5 per group) were infected with the ASFV-ΔECM3 virus or the WT virus intramuscularly (IM), both at a dose of 10^5^ TCID_50_. ASFV-inoculated pigs were monitored for rectal temperature as described previously (Liu et al., 2021). Clinical samples of sera, heparin-anticoagulated whole blood, oral swabs, nasal swabs, and anal swabs were collected at 0, 3, 7, 10, 14, 21, and 28 dpi. The pigs were euthanized by injection of sodium pentobarbital and then subjected to necropsy examination at 28 dpi. To assess the stability and virulence of the recombinant mutant virus, the ASFV-ΔECM3 virus was passaged serially in pigs five times. The organs and tissues of ASFV-ΔECM3 virus-infected pigs euthanized were collected, and the viral distribution in pigs was analyzed. Organs and tissues from the dead pigs and surviving pigs that were euthanized at the end of the observation period were scored using the gross scoring system, and 100 mg of the indicated samples were homogenized by Tissuelyser-FEII (Shanghai Jingxin Co. Ltd., Shanghai, China) in 800 μl PBS supplemented with 1% Pen-strep for the detection of virus titer by TCID_50_ assay as described previously (Liu et al., 2021).

#### Experiment 2

Efficacy evaluation of protection by vaccination with ASFV-ΔECM3 in pigs. To investigate the immunogenicity of the ASFV-ΔECM3 virus in pigs, 10 piglets were divided into two groups. Five pigs were immunized with 10^5^ TCID_50_ of the ASFV-ΔECM3 virus. At 28 dpi, the non-immunized group (*n* = 5) and the immunized group (*n* = 5) were IM challenged with 10^2^ HAD_50_ of the virulent parental GZ201801 virus and later subjected to necropsy examination at 75 days post-challenge (dpc). To evaluate pathological changes in pigs immunized with ASFV-ΔECM3, spleen, lung, liver, kidney, and mandibular lymph node tissues were collected and stained with H & E stain and TUNEL assay. The samples were collected and determined following the methods mentioned earlier.

### Quantitative PCR

African swine fever virus genomic DNA was extracted from tissue homogenate (Tissuelyser-FEII, Shanghai, China) and whole blood using GlinX Viral Nucleic Acid Extraction kits (GlinX, Shanghai, China). The qPCR procedure was carried out on a QuantStudio 5 system (Applied Biosystems, USA) according to the protocol of ASFV dtec-qPCR test kits (Genetic PCR Solutions, Spain).

### Analysis of Immune Response to ASFV-ΔECM3

The ID Screen^®^ African Swine Fever Competition ELISA kits (IDVet Innovative Diagnostics Louis Pasteur, Grabels, France) were used to detect specific anti-ASFV antibodies in serum. For each sample, the competition percentage (S/N%) was calculated according to the manufacturer's instructions. Less than or equal to 40% was considered positive, between 40 and 50% was considered doubtful, and ≥50% was considered negative.

### Biosafety Statement and Facility

Animal experiments were performed under Animal Biosafety Level 3 (ABSL-3) conditions in The Spirit Jinyu Biological Pharmaceutical Co., LTD and approved by the China National Accreditation Service for Conformity Assessment (CNAS) with a license of CNAS-BL0101. The protocols of ASFV infection were approved by the Ministry of Agriculture and Rural Affairs.

### Statistical Analysis

Statistical analyses were performed using unpaired, two-tailed Student's *t*-test. A *p*-value < 0.05 was considered statistically significant.

## Results

### Development of ASFV-ΔECM3 Deletion Mutant

**Figure 1:**
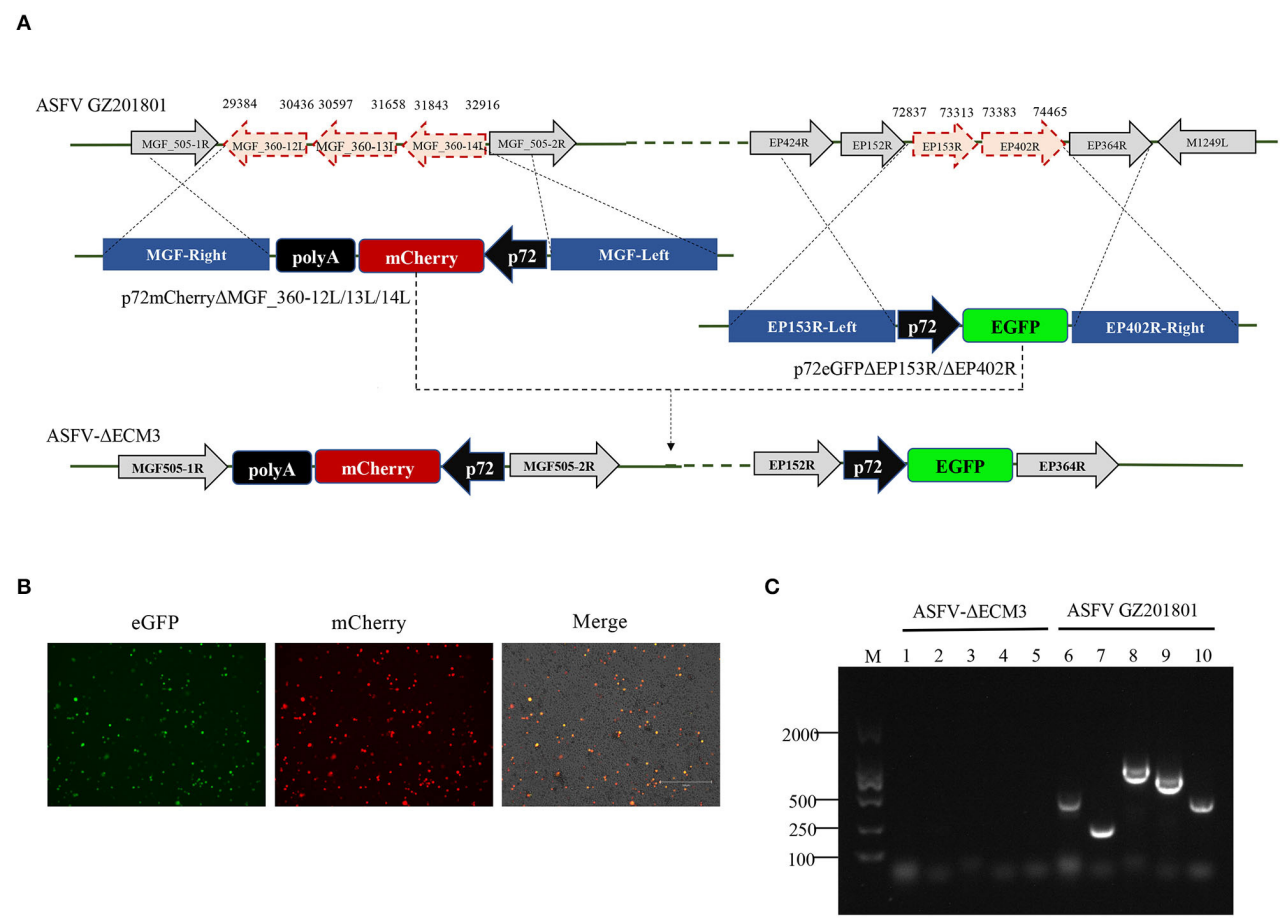
Schematic of the construction of ASFV-ΔECM3 deletion mutant. **(A)** The *EP153R, EP402R, MGF_360-12L, MGF*_*360-13L*, and *MGF*_*360-14L* genes from the GZ201801 genome were replaced by the p72eGFPΔEP153R/ΔEP402R and p72mCherryΔMGF_360-12L/13L/14L recombination transfer vectors by homologous recombination. **(B)** eGFP and mCherry reporter fluorescence indicate BMDM cells infection with the ASFV-ΔECM3 virus. **(C)** Confirmation of the ASFV-ΔECM3 deletion mutant by PCR. Lanes 1 and 6 test for the *EP153R* gene. Lanes 2 and 7 test for the *EP402R* gene. Lanes 3 and 8 test for the *MGF_360-12L* gene. Lanes 4 and 9 test for the *MGF*_*360-13L* gene. Lanes 5 and 10 test for the *MGF*_*360-14L* gene.

Deletion of the *EP153R*-*EP402R* genes from 72,837–74,465 nt in the viral genome was achieved by homologous recombination using the p72eGFPΔEP153R/ΔEP402R vector (Figure 1A). The *EP153R-EP402R* genes were replaced by a p72eGFP expression cassette. Based on this deletion mutant, second gene loci from 29,384–32,916 nt including the *MGF_360-12L, MGF_360-13L*, and *MGF_360-14L* genes was replaced with a p72mCherry expression cassette with the p72mCherryΔMGF_360-12L/13L/14L vector by homologous recombination. The deletion mutant was designated as ASFV-ΔECM3 (Figure 1A). The ASFV-ΔECM3 virus grew easily in cells with a high titer of more than 10^7^ TCID_50_/ml. BMDMs were infected with the purified ASFV-ΔECM3 virus, showing stable expression of eGFP and mCherry reporter protein (Figure 1B). Following several rounds of purification, PCR confirmation was performed to verify the deletion of these five genes (Figure 1C). Compared with the WT virus, no bands were amplified from the ASFV-ΔECM3 virus. The accuracy of the ASFV-ΔECM3 virus genome was further confirmed by next-generation sequencing.

### Replication of ASFV-ΔECM3 in BMDMs

**Figure 2:**
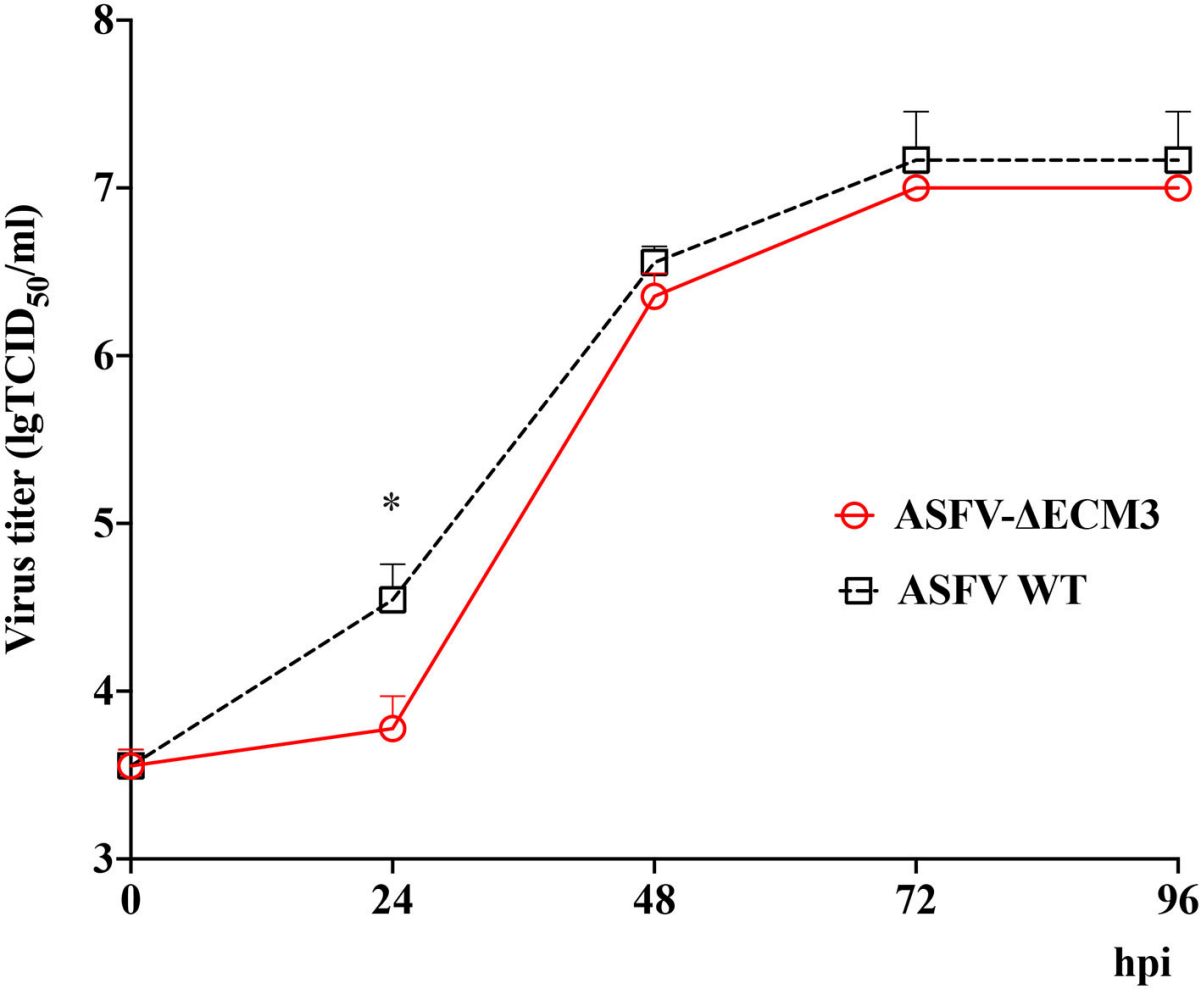
*In vitro* growth characteristics of ASFV-ΔECM3 and parental GZ201801 virus. Primary swine macrophage cell cultures were infected (MOI = 0.01) with either virus, and virus yield was titrated at the indicated times post-infection. Data represent means and SD from three independent experiments. ^*^p < 0.05.

Cells and supernatants were collected at different time points post-infection. The results showed that the ASFV-ΔECM3 virus displayed a growth kinetic similar to that of the parental GZ201801 WT virus (Figure 2). The growth titer of the ASFV-ΔECM3 virus was slower than that of the WT virus at 24 hpi. The mean titer of the ASFV-ΔECM3 virus was 6.00 × 10^3^ TCID_50_/ml, while that of the WT virus was 3.51 × 10^4^ TCID_50_/ml, about 6-fold less. Until 96 hpi, the titer of the ASFV-ΔECM3 virus was 1.00 × 10^7^ TCID_50_/ml, at which time the titer of the WT virus was 1.47 × 10^7^ TCID_50_/ml. Thus, the deletion of the *EP153R* and *EP402R* or *EP153R, EP402R, MGF_360-12L, MGF_360-13L*, and *MGF_360-14L* genes did not significantly affect the replication ability of the GZ201801 virus in swine macrophages.

### Safety Investigation of ASFV-ΔECM3 Virus in Pigs

**Figure 3:**
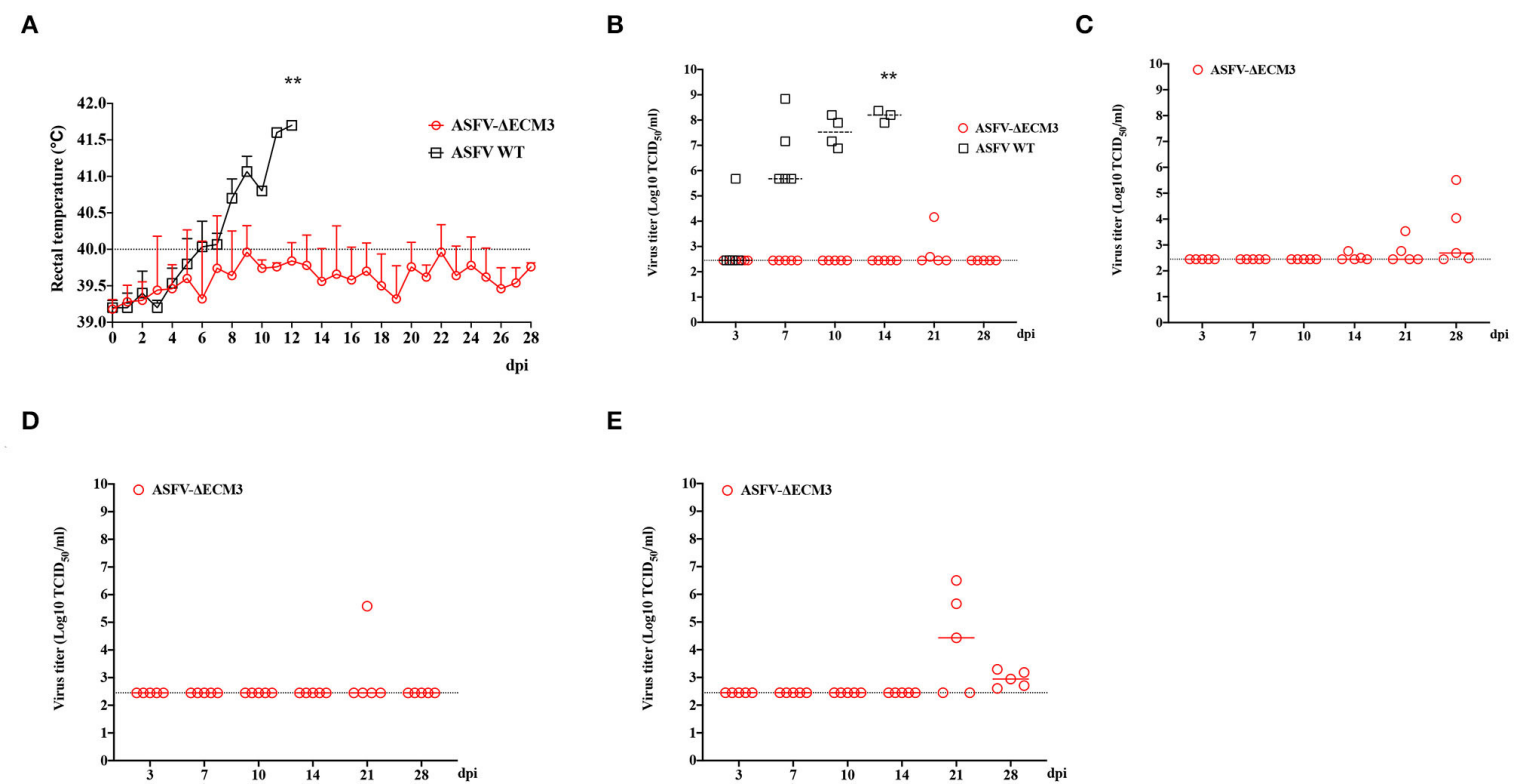
Safety investigation of ASFV-ΔECM3 as a vaccine candidate in pigs. **(A)** Body temperature in piglets challenged with ASFV-ΔECM3 virus and parental GZ201801. **(B)** Viremia titers were detected in piglets inoculated with ASFV-ΔECM3 virus. Viral titers of oral swabs **(C)**, nasal swabs **(D)**, and anal swabs **(E)** were evaluated post-infection. The sensitivity of virus detection was ≥ 10^2.45^ TCID_50_/ml, which meant Cycle threadhold (Ct) more than 35 might be considered below the limit of detection by qPCR analysis. ^**^p < 0.01.

To determine the pathogenicity of the virus as a live-attenuated vaccine candidate *in vivo*, five pigs were inoculated with the ASFV-ΔECM3 virus. None of the piglets had remarkable clinical signs in the group of the ASFV-ΔECM3 virus. All of them survived until 28 dpi. The body temperatures of the five piglets were below 40.0°C, with no significant increase during the examination period. However, all the piglets in the WT virus group showed a rapid increase after 7 dpi (Figure 3A).

**Figure 4:**
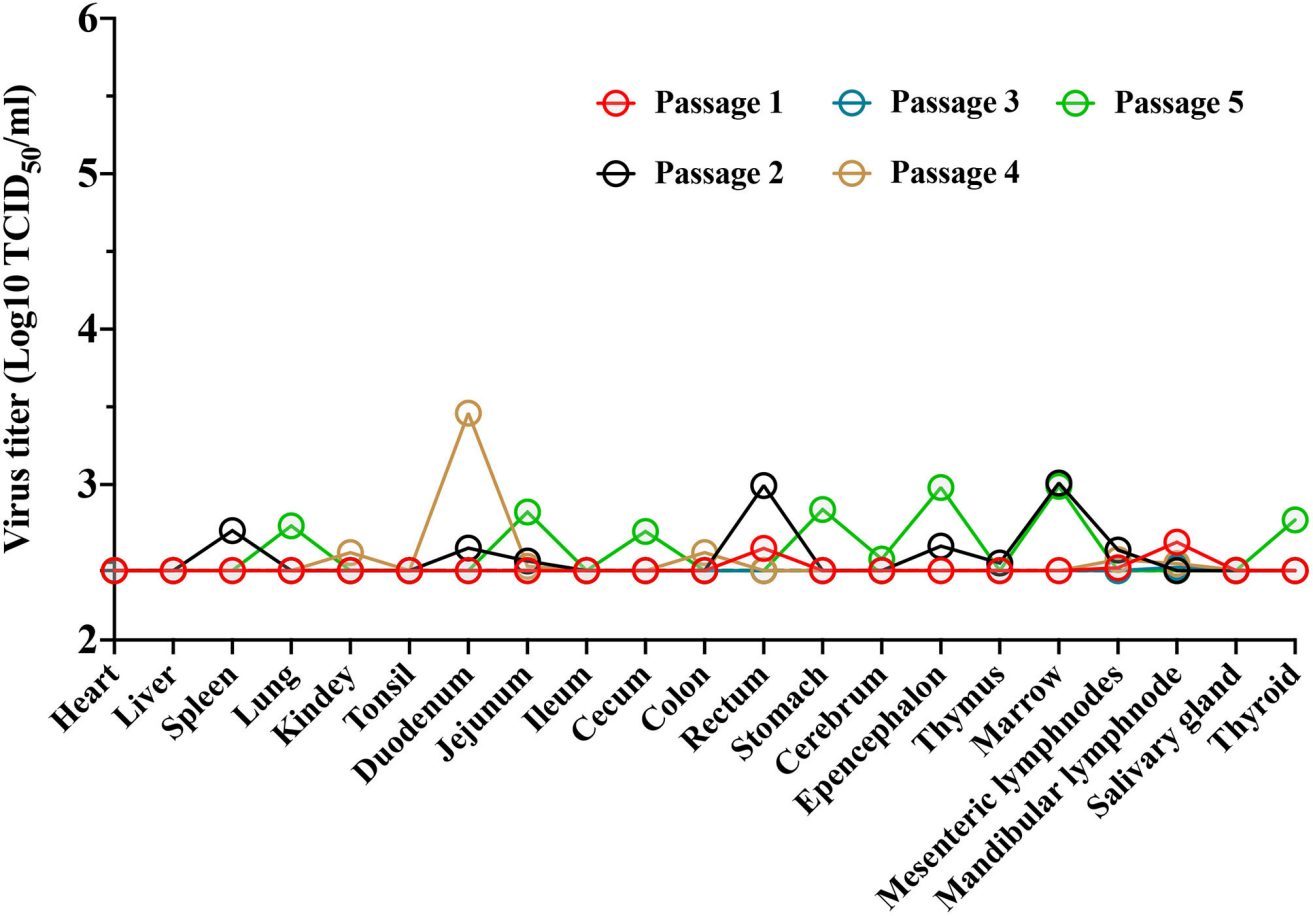
Virulence of the ASFV-ΔECM3 virus by serial passages in pigs. The ASFV-ΔECM virus was serially passaged in pigs five times, the organs and tissues with lesions were collected, dissociated, and re-injected into pigs. The qPCR analysis for detection of the viral distribution in pigs. The sensitivity of virus detection was ≥ 10^2.45^ TCID_50_/ml.

Blood samples were collected at 0, 3, 7, 10, 14, 21, and 28 dpi to measure ASFV genomic DNA expression in whole blood. Only two of the five pigs in the group of animals infected with ASFV-ΔECM3 showed slightly low-virus shedding in blood with titers of 1.46 × 10^4^ TCID_50_/ml and 0.39 × 10^3^ TCID_50_/ml, respectively. The mean titer of the group was 0.66 × 10^3^ TCID_50_/ml at 21 dpi (Figure 3B). Viral DNA was detectable in oral swabs (Figure 3C), nasal swabs (Figure 3D), and anal swabs (Figure 3E) beginning at 14 dpi, and the virus titers in the oral swabs and anal swabs were much higher than those in the nasal swabs. In addition, the ASFV-ΔECM3 virulence was preserved by serial passages in pigs (Figure 4). It showed that the ASFV-ΔECM3 virus was less virulent to pigs and that serial deletions of the *EP153R, EP402R, MGF_360-12L, MGF_360-13L*, and *MGF_360-14L* genes improved the safety of the vaccine candidate.

### Efficacy Evaluation of Protection by Vaccination With ASFV-ΔECM3 in Pigs

**Figure 5:**
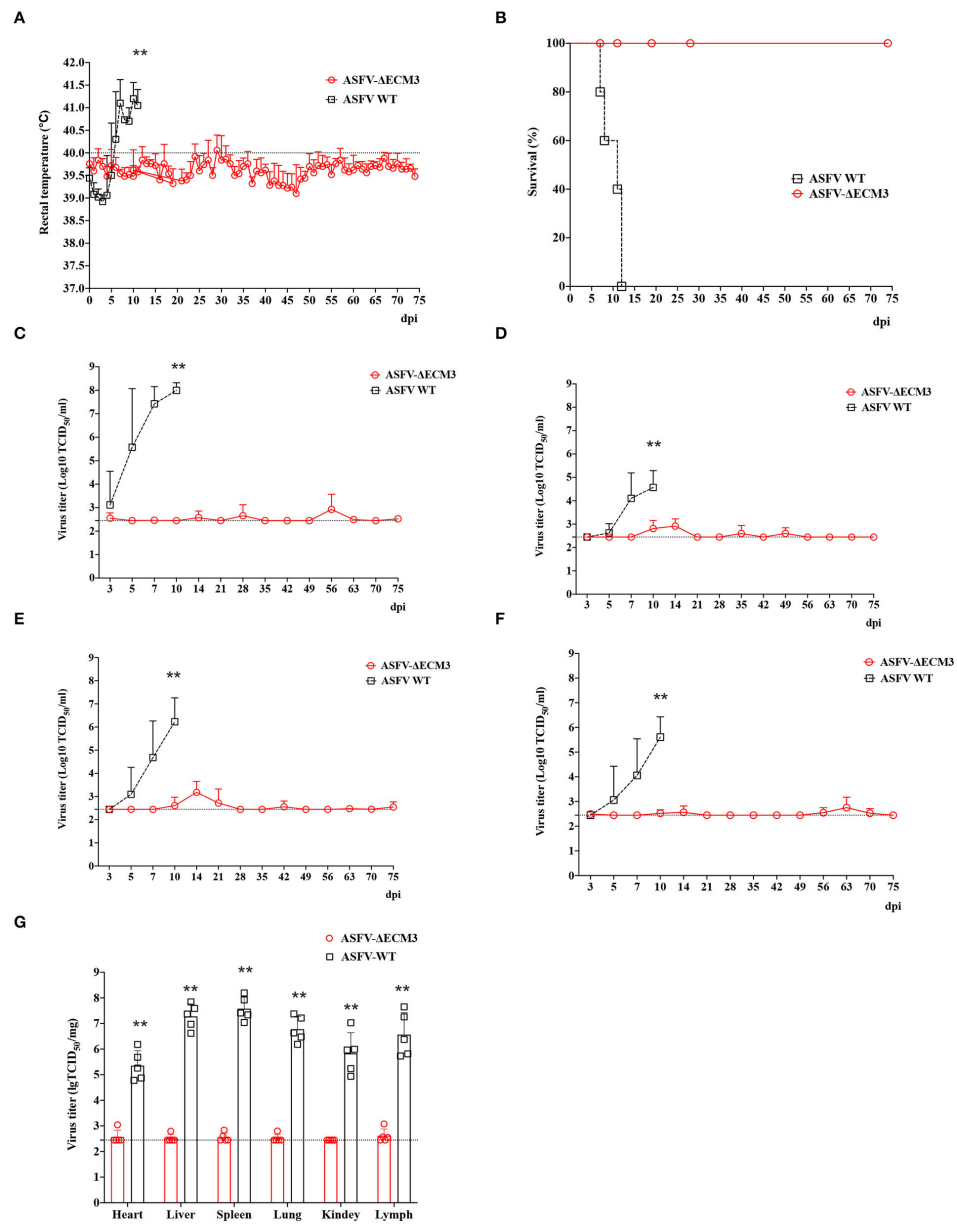
The efficacy of ASFV-ΔECM3 virus against ASFV-GZ201801 infection. **(A)** Body temperature in the ASFV-ΔECM3 virus immunized piglets challenged with parental GZ201801. **(B)** Percentage of animals surviving after challenge. **(C)** Viremia titers were detected in piglets that were immunized with ASFV-ΔECM3 virus after being challenged with the parental GZ201801 virus. Viral titers of oral swabs **(D)**, nasal swabs **(E)**, and anal swabs **(F)** were detected in piglets that were immunized with ASFV-ΔECM3 virus after being challenged with parental GZ201801 virus. **(G)** The viral shedding in the tissues was measured. The sensitivity of virus detection was ≥10^2.45^ TCID_50_/ml. ^**^p < 0.01.

**Figure 6:**
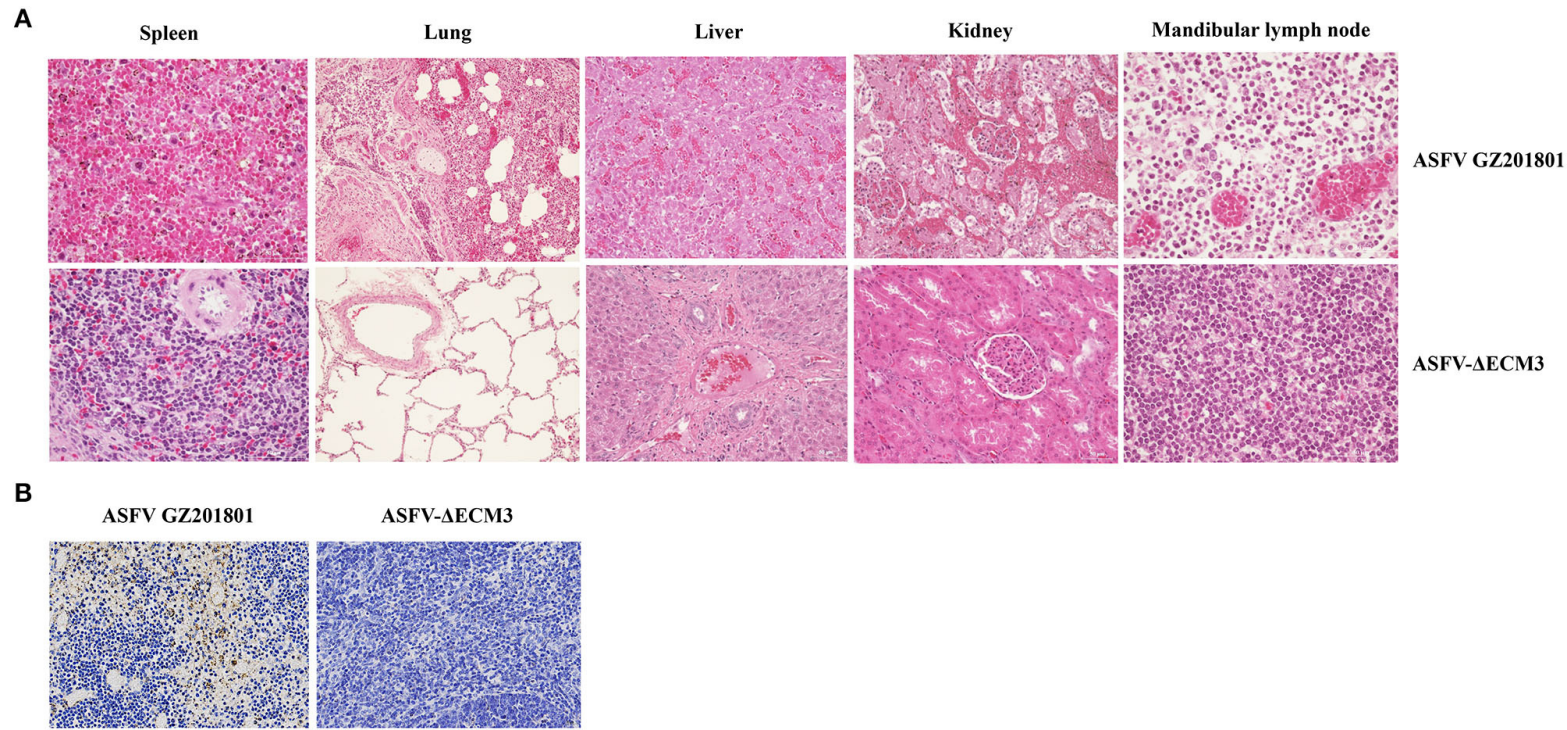
Histological sections of the tissues by HandE staining and TUNEL assay. **(A)** Histopathological analysis of the spleen, lung, liver, kidney, and mandibular lymph node in the ASFV-ΔECM3 virus and parental GZ201801 (H and E staining). **(B)** TUNEL assay of lymph nodes.

To evaluate the protective efficacy of ASFV-ΔECM3 as a potential live-attenuated vaccine, all the piglets were challenged with 10^2^ HAD_50_ of virulent parental GZ201801 at 28 dpi. The results showed that all the piglets of the ASFV-ΔECM3-vaccinated group survived with a transient temperature increase period (Figure 5A). However, the rectal temperatures in all the piglets of the virulent parental GZ201801 WT virus group increased rapidly after 5 dpi. All the animals showed classical lesions at 12 dpi without survival (Figure 5B). Viral shedding of the piglets in the ASFV-ΔECM3 virus-immunized group was very low or undetectable, at <6.17 × 10^3^ TCID_50_/ml. However, viremia was much higher at up to 6.92 × 10^8^ TCID_50_/ml in the WT virus group (Figure 5C). After the challenge, high virus titers were detected in the oral swabs (Figure 5D), nasal swabs (Figure 5E), and anal swabs (Figure 5F) from all pigs in the virulent parental GZ201801 WT virus group, while pigs inoculated with ASFV-ΔECM3 pigs had either very low or undetectable viral titers. As expected, the pigs infected with the parental ASFV WT virus exhibited an increased body temperature (>41.36°C) at 3.6 dpi (Table 2). At the end of the experiment, the piglets were necropsied, and tissue samples were collected from the hearts, kidneys, livers, lungs, and lymph nodes. Compared with the high level of virus shedding in the WT virus group, viral shedding in the tissues was very limited, below a level of 1.10 × 10^3^ TCID_50_/ml in the ASFV-ΔECM3 virus-immunized group (Figure 5G). Lesions on spleens showed more severe bleeding and splenic lymphocytes exhibited more variable degrees of necrosis in the GZ201801 virus group than in the ASFV-ΔECM3 virus group (Figure 6A). Lesions on the lung showed pulmonary interstitial widening and hyperemia. Liver pathological changes were seen in the liver interstitial widening and hepatic sinusoid congestion (Figure 6A). The kidney exhibited slight pathological changes (Figure 6A). Mandibular lymph nodes showed more prominent congestion in the GZ201801 virus group than in the ASFV-ΔECM3 virus group (Figure 6A). By TUNEL assay, in the lymph nodes, no cells were positively stained in the ASFV-ΔECM3 virus group (Figure 6B). The fragmented DNAs significantly revealed many apoptotic cells in the GZ201801 virus group. These results indicate that the ASFV-ΔECM3 deletion mutant almost completely protected pigs against homologous virulent challenge.

**Table 2:** Swine survival and fever response following infection with ASFV-ΔECM3 and challenge with parental GZ201801.

**Virus**	**No. of survivors** **(*n* = 5)**	**Time to death** **[Mean (SD)] (days)**	**Data for fever [mean (SD)]**		
			**No. of days to onset**	**Duration (days)**	**Maximum daily temp (**°**C)**
ASFV-GZ201801	0	10	6	3.6	41.36^*^
ASFV-ΔECM3	5	-	-	-	39.74

* *p < 0.05*.

### Analysis of Immune Response to ASFV-ΔECM3 in Pigs

**Figure 7:**
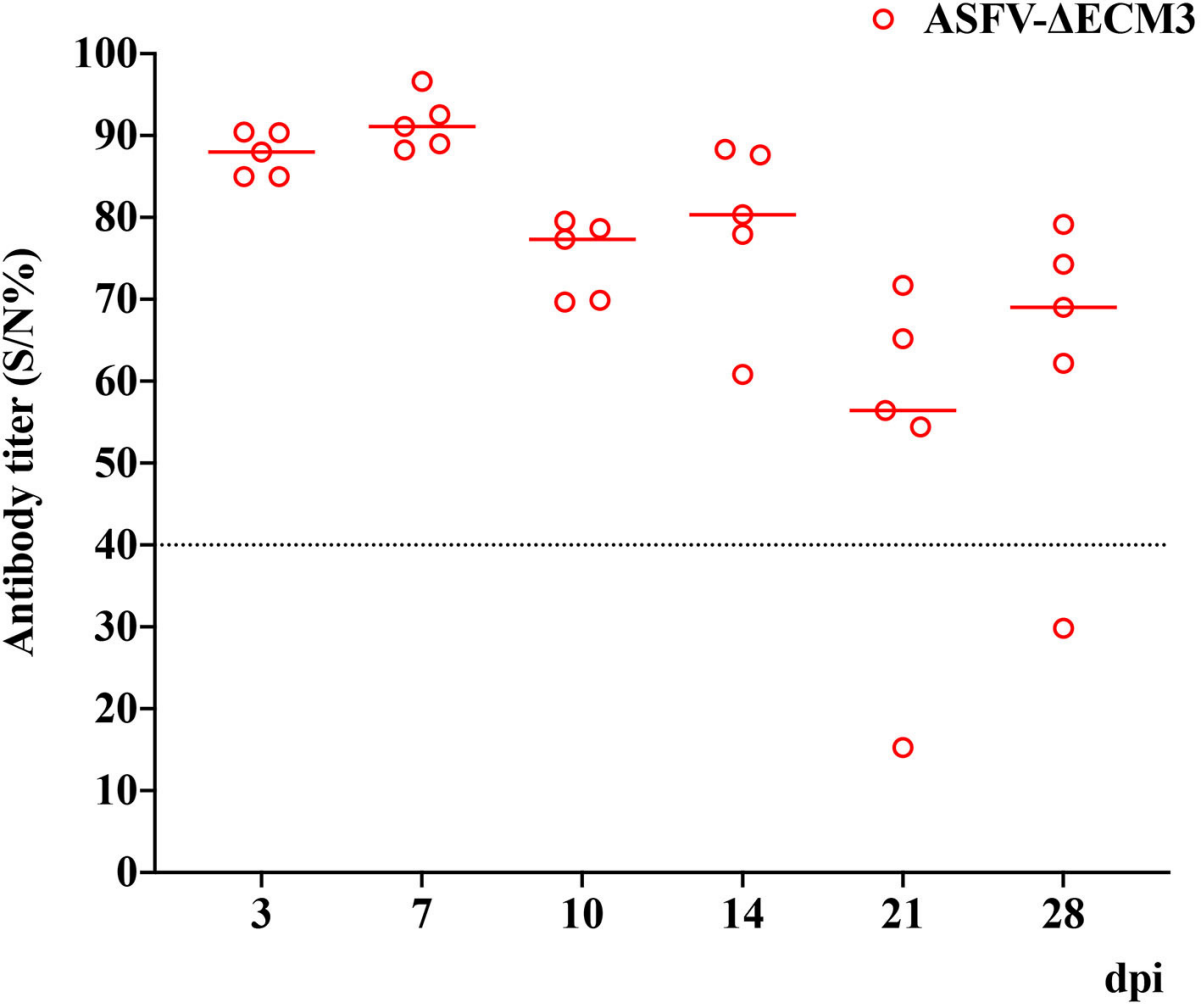
Detection of antibodies against ASFV in sera of pigs. Serum levels of ASFV-specific antibodies were measured using the African Swine Fever Competition ELISA kits. Positive: S/N%≤40%. Doubtful: 40%< S/N% <50%. Negative: S/N% ≥50%.

Serum levels of specific anti-ASFV antibodies were evaluated by ELISA in the ASFV-ΔECM3 group during the immunization (Figure 7). However, among the five pigs infected with ASFV-ΔECM3, only one pig produced specific anti-ASFV antibodies. It was assumed that due to the contiguous deletion encompassing the *EP153R* and *EP402R* genes, the production of the specific anti-ASFV antibodies tended to appear delayed and lower.

## Discussion

ASF has been causing significant economic losses to the global swine industry. To prevent ASFV infection, several vaccine technologies have been developed, including those based on live attenuated, inactivated, subunit, vectored, and DNA vaccines (Sang et al., 2020). Live-attenuated vaccines have been shown to produce a high level of protection by targeted gene deletion. For example, the deletion of the *I177L* or *DP148R* genes protected the swine against homologous virulent challenge (Reis et al., 2017; Borca et al., 2020).

Different combinations of deletions can affect vaccine efficacy. Previous research has shown that the different gene deletion combinations of *MGF_360, MGF_505, CD2v, UK*, and *9GL* provide reliable protection against homologous virulent viruses (O'Donnell et al., 2016b; Chen et al., 2020; Teklue et al., 2020). In contrast, the simultaneous deletion of the *MGF_360/505* and *9GL* genes reduces its protective effect (O'Donnell et al., 2016a). Although live-attenuated vaccines have been shown to be highly effective, they raise safety concerns, such as the presence of persistent low-level viremia and fever have been reported. Recent studies have shown that the deletion of the *EP153R* and *EP402R* genes from BeninΔDP148 plays a synergistic role in reducing clinical signs and levels of virus in the blood (Petrovan et al., 2022). In any case, further studies are necessary to determine which genes may increase the safety and efficacy of live-attenuated vaccines.

Here, we constructed a deletion mutant ASFV-ΔECM3 of GZ201801 by deleting the *EP153R, EP402R, MGF_360-12L, MGF_360-13L*, and *MGF_360-14L* genes. After immunization with the ASFV-ΔECM3 vaccine, 100% of the pigs survived, and no clinical signs were observed. The normal body temperature of the ASFV-ΔECM3-immunized pigs was maintained, while that of those infected with ASFV-GZ201801 was not. However, in the group of animals vaccinated with ASFV-ΔECM3, only one pig produced specific anti-ASFV antibodies, and the production appeared delayed and lower. This may have been due to the contiguous deletion encompassing the *EP153R* and *EP402R* genes, as previously reported in BeninΔDP148RΔEP402RΔEP153R (Petrovan et al., 2022). The histopathology results suggested that lesions on the spleen, lung, liver, kidney, and mandibular lymph nodes were more severe in the ASFV-GZ201801-infected pigs than in the ASFV-ΔECM3-immunized pigs. Our results suggested that the deletions in the *EP153R-EP402R, MGF_360-12L-14L* regions of the ASFV genome decreased the pathogenicity significantly and increased safety. Importantly, animals infected with ASFV-ΔECM3 were effectively protected against homologous virulent challenge.

Viremia causes systemic virus dissemination and shedding of virus, and viremia titers are the best correlates of safety in live-attenuated vaccines. In this study, we observed that the pigs had limited viral shedding in blood. The pigs vaccinated with the ASFV-ΔECM3 virus either had very low level or undetectable viral titers in the oral swabs, nasal swabs, and anal swabs. This may have been due to the correlation between the viremia and the ASFV *EP402R* gene, as previous studies have indicated (Borca et al., 1998). Thus, we constructed a deletion mutant ASFV-ΔECM3 by deleting the *MGF_360-12L, MGF_360-13L*, and *MGF_360-14L* genes. The contiguous deletion encompassing the *EP153R* and *EP402R* genes attenuated the virulence of the live-attenuated vaccine. In the future, we will target more genes target for recombination to delete more functional genes based on the ASFV-ΔECM3 vaccine candidate to balance the virulence and safety, further analyzing the effect of deletion mutations on live-attenuated vaccines.

## Data Availability Statement

The sequencing data have been deposited in the NCBI Sequence Read Archive (SRA) repository, accession number: SRR20637402.

## Ethics Statement

The animal study was reviewed and approved by Spirit Jinyu Company.

## Author Contributions

HC and HW conceived and designed the experiments. ZX, YLiu, DD, JL, LL, LG, ZC, XL, and HC performed the experiments. YLi, WY, and QZ analyzed the data. HC and QS contributed reagents/materials/analysis tools. ZX, JL, HW, and HC wrote the paper. All authors contributed to the article and approved the submitted version.

## Funding

This work was supported by the National Natural Science Foundation of China (Grant Nos. 32170161 and U19A2039), the National Key Research and Development Program of China (Grant Nos. 2021YFD1801401 and 2021YFD1801300), and the Key-Area Research and Development Program of Guangdong Province (2019B020211003).

## Conflict of Interest

DD and QS were employed by the Spirit Jinyu Biological Pharmaceutical Co., Ltd. The remaining authors declare that the research was conducted in the absence of any commercial or financial relationships that could be construed as a potential conflict of interest. The reviewer H-JQ declared a shared affiliation with the authors HC, ZX, YLi, JL, ZC, YLiu, WY, LL, QZ, YS, and XL at the time of the review. The handling editor XY declared a shared affiliation with the authors HC, ZX, YLi, JL, ZC, YLiu, WY, LL, QZ, YS, and XL at the time of the review.

## Publisher's Note

All claims expressed in this article are solely those of the authors and do not necessarily represent those of their affiliated organizations, or those of the publisher, the editors and the reviewers. Any product that may be evaluated in this article, or claim that may be made by its manufacturer, is not guaranteed or endorsed by the publisher.
